# Past, Present, and Future of Gastrointestinal Microbiota Research in Cats

**DOI:** 10.3389/fmicb.2020.01661

**Published:** 2020-07-24

**Authors:** Yang Lyu, Chunxia Su, Adronie Verbrugghe, Tom Van de Wiele, Ana Martos Martinez-Caja, Myriam Hesta

**Affiliations:** ^1^Department of Nutrition, Genetics and Ethology, Faculty of Veterinary Medicine, Ghent University, Merelbeke, Belgium; ^2^Department of Green Chemistry and Technology, Faculty of Bioscience Engineering, Ghent University, Ghent, Belgium; ^3^Department of Clinical Studies, Ontario Veterinary College, University of Guelph, Guelph, ON, Canada; ^4^Center of Microbial Ecology and Technology, Faculty of Bioscience Engineering, Ghent University, Ghent, Belgium

**Keywords:** microbiome, gastrointestinal tract, molecular techniques, nutrition and diseases, feline

## Abstract

The relationship between microbial community and host has profound effects on the health of animals. A balanced gastrointestinal (GI) microbial population provides nutritional and metabolic benefits to its host, regulates the immune system and various signaling molecules, protects the intestine from pathogen invasion, and promotes a healthy intestinal structure and an optimal intestinal function. With the fast development of next-generation sequencing, molecular techniques have become standard tools for microbiota research, having been used to demonstrate the complex intestinal ecosystem. Similarly to other mammals, the vast majority of GI microbiota in cats (over 99%) is composed of the predominant bacterial phyla *Firmicutes*, *Bacteroidetes*, *Actinobacteria*, and *Proteobacteria*. Many nutritional and clinical studies have shown that cats’ microbiota can be affected by several different factors including body condition, age, diet, and inflammatory diseases. All these factors have different size effects, and some of these may be very minor, and it is currently unknown how important these are. Further research is needed to determine the functional variations in the microbiome in disease states and in response to environmental and/or dietary modulations. Additionally, further studies are also needed to explain the intricate relationship between GI microbiota and the genetics and immunity of its host. This review summarizes past and present knowledge of the feline GI microbiota and looks into the future possibilities and challenges of the field.

## Introduction

The intestinal microbiome is a complex collection of microorganisms (i.e., bacteria, archaea, viruses, fungi, and protozoa) (Frank et al., 2007). Based on the study of the small ribosomal subunit RNA (16S rRNA), current phylogenetic research has demonstrated that the mammalian gastrointestinal (GI) tract harbors hundreds to thousands of microbial phylotypes (Suchodolski et al., 2009). According to recent reports, approximately 10^10^ to 10^14^ microbes are present in the mammals’ GI tract (Frank et al., 2007), which is around ten times the total amount of host cells (Hoffmann et al., 2016). This complex system is composed of the mutual interaction between host cells and resident microorganisms and is known as the gastrointestinal microbiome (Suchodolski, 2011a).

**FIGURE 1:**
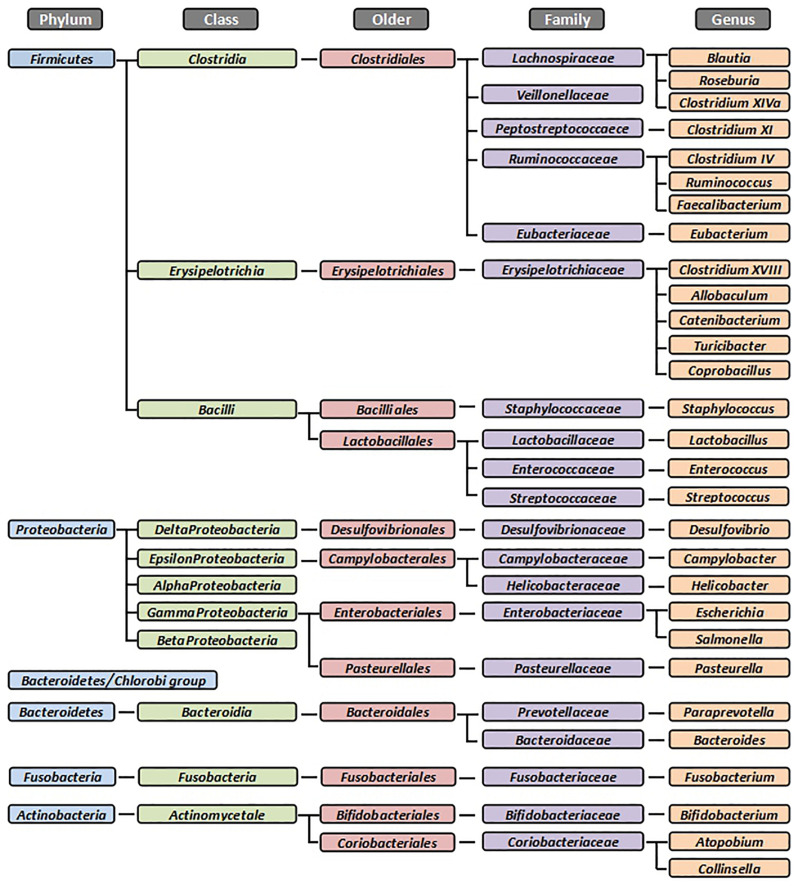
The dominant bacterial groups in the feline gastrointestinal tract. Summarized from Osbaldiston and Stowe (1971), Johnston et al. (1993, 2000, 2001), Inness et al. (2007), Desai et al. (2008), Janeczko et al. (2008), Ritchie et al. (2008), Abecia et al. (2010), Barry et al. (2010, 2012), Garcia-Mazcorro et al. (2011), Jia et al. (2011a, b), Sparkes et al. (1998b), and Tun et al. (2012). All bacteria were identified from fecal samples or intestinal biopsies by the molecular method.

Domestic cats (*Felis catus*) are an obligate carnivore which depend on high intakes of animal tissue to meet its nutrition requirements. This has led to a metabolic adaptation to a low-glucose and high-protein metabolism (Macdonald et al., 1984; Verbrugghe et al., 2012). Compared to humans or other mammals, cats are less dependent on the intestinal microbiota for energy acquisition through microbial fermentation. Nevertheless, a stable and balanced microbiota remains critical for the maintenance of intestinal health (Deng and Swanson, 2015). Similarly to other mammals, the dominant bacterial phyla in the feline GI tract are *Firmicutes*, *Bacteroidetes*, *Proteobacteria*, and *Actinobacteria* (Figure 1). However, according to the literature (Suchodolski, 2011a; Minamoto et al., 2012; Barko et al., 2018; Tizard and Jones, 2018), the percentages of these bacterial groups often differ among species and individuals. These variations may be caused by the animals’ living environment or by the different experimental methods used (Deng and Swanson, 2015).

Research on the GI microbiota of cats is a field in permanent expansion. Recently developed molecular techniques have improved the knowledge on the composition, alterations, and function of the feline gastrointestinal ecosystem (Desai et al., 2008; Ritchie et al., 2008; Swanson et al., 2011). Several exogenous factors (e.g., diet) have been demonstrated to influence the microbiota composition to some extent (Desai et al., 2008; Ritchie et al., 2008; Barry et al., 2010; Middelbos et al., 2010; Swanson et al., 2011). Nevertheless, the microbiota is resilient to most environmental influences, rapidly returning to its pretreatment state (Middelbos et al., 2010). Several studies on humans and dogs have reported that the administration of antibiotics could cause more profound shifts of the GI microbiota composition, and some bacterial groups could remain depressed for several weeks (Dethlefsen et al., 2008; Suchodolski et al., 2009; Torres-Henderson et al., 2017; Whittemore et al., 2018, 2019). Recent evidence both in humans and in animals including cats has already shown implicating alterations in the composition of the GI microbiota to chronic enteropathies (Inness et al., 2007; Janeczko et al., 2008). Moreover, extraintestinal disorders, such as atopic dermatitis, have been associated with GI dysbiosis due to the mutual interaction between GI microbiome and host immunity (Penders et al., 2007). These results highlight the importance of maintaining the balance of the gastrointestinal ecosystem.

This review focuses on the feline GI microbiota and summarizes the past and present knowledge on the GI microbiota in cats, including characterization techniques, composition, roles in health and disease, effects to different treatments, and future directions. All citations in this review were obtained from the online open database Google Scholar,^1^ the National Centre for Biotechnology Information (NCBI),^2^ and ScienceDirect,^3^ using search terms “microbiota/microbiome in cats” or “feline microbiota/microbiome,” within the time frame from 1990 to the present.

## Composition

### Result From Traditional Cultivation Methods

At the early stages of microbial research, traditional cultivation techniques were the most common method to characterize intestinal microbiota. Many pioneer researchers explored this area using culture-based methods and observed the bacterial composition of intestinal and fecal samples in cats. The most abundant cultivable groups found were *Bacteroides*, *Clostridium*, *Enterococcus*, *Streptococcus*, *Fusobacteria*, and *Eubacteria* (Osbaldiston and Stowe, 1971; Terada et al., 1993; Sparkes et al., 1998a, b; Johnston et al., 2000, 2001). Similarly to humans and other species, the abundance of microbiota in the cat’s intestine increases along the gut (Handl et al., 2011; Deng and Swanson, 2015). The proximal intestine contains a practically equal distribution of aerobic and anaerobic bacteria, while distal portions are predominantly colonized by anaerobic bacterial groups (Minamoto et al., 2012). Interestingly, some culture-based studies have suggested that, compared to bacterial counts in the small intestine of dogs and humans, cats display a relatively higher number of bacteria, especially anaerobic (Johnston et al., 1993; Greetham et al., 2002). Nevertheless, phenotypical and biochemical characterization systems have often failed to precisely identify many microbes present in the GI tract (Suchodolski, 2011a; Deng and Swanson, 2015). Hence, DNA sequencing of cultured isolations was often required, which have given a vigorous boost to the development of today’s molecular techniques (Suchodolski, 2011b).

### Results From Molecular Techniques

The variable region of the 16S rRNA gene of bacteria contains the signature of a phylogenetic group and even species, based on the revelation of this knowledge; many new tools for analysis of microbial community became available (Tun et al., 2012). In the last 20 years, several molecular tools have facilitated a more in-depth characterization of the complex intestinal microbiota (Tannock, 2005). These tools have now largely replaced traditional bacterial culture methods, becoming the standard approach to study the microbial ecology (Suchodolski et al., 2008). Current molecular techniques include fluorescence *in situ* hybridization (FISH), polymerase chain reaction (PCR, i.e., PCR/DGGE, qPCR), and sequencing (i.e., 454-pyrosequencing, shotgun sequencing) (Tannock, 2005; Suchodolski et al., 2009, 2015; Swanson et al., 2011); details are summarized in Table 1. Molecular techniques allow the characterization of unidentified gastrointestinal microorganisms in the past (Swanson et al., 2011). Combining them with metagenomics tools, these techniques can also provide description of the functional potentials of microbiota (Tun et al., 2012).

**TABLE 1:** Frequently used methods in feline microbiota studies.

Techniques	Purpose	Summary
FISH	Detection and quantification of bacterial cells	Fluorescent dye-labeled oligonucleotide probe hybridizes to ribosomal RNA sequence in cells fixed on slides with wells. Enumeration by epifluorescence microscopy
PCR/DGGE	Profiling the composition of bacterial communities for comparative analysis	Separation of 16S rDNA fragments from different bacterial types is based on differences in chemical stability, through a linearly increasing gradient of chemical denaturants. The profile of DNA fragments represents the genetic fingerprint of the community.
Qpcr	Quantification of bacteria	PCR primers and a labeled probe (often incorporating a reporter dye and a quencher molecule) are used to measure the real-time accumulation of a specific target sequence
454-Pyrosequencing	Detecting the nucleotide incorporated	A single strand of DNA is used as a template to synthesize the sequence of its complementary strand, which is determined by a chain of reactions resulting in light being emitted when a specific nucleotide or length of nucleotides are added to the complementary sequence
Shotgun sequencing	Determining the sequence of entire chromosomes and genomes	Based on producing random fragments of DNA that are then assembled by computers that order fragments by finding overlapping ends

*FISH, fluorescence in situ hybridization; PCR/DGGE, polymerase chain reaction combined with denaturing gradient gel electrophoresis; qPCR, real-time quantitative polymerase chain reaction. Summarized from Tannock (2005), Suchodolski et al. (2009, 2015), Suchodolski (2011b), and Swanson et al. (2011).*

#### Fish

Studies using FISH have indicated that the total count of bacteria present in the cat’s intestine is approximately 10.5 log_10_ cells/g feces (Minamoto et al., 2012). The most abundant populations in intestines of young (1–3 years old) and senior (8–14 years old) cats belong to the *Atopobium* group (probe Ato291) – including *Coriobacteriaceae*, *Clostridium cluster XIVa* – and lactic acid bacteria, including *Bifidobacteria* (Abecia et al., 2010; Jia et al., 2011a, b). Studies on fecal samples from adult cats have shown similar results, as well as feces of cats with inflammatory bowel diseases (IBD) (Inness et al., 2007; Abecia et al., 2010). However, the total count of *Bifidobacteria* in healthy adult cats varied between two studies (approximately 11% vs. 30%). FISH targets 16S rRNA, diametrically quantifying bacteria using fluorescent-labeled probes. Nevertheless, this tool is too labor intensive, and the probes need to be designed specifically for a particular bacterial group (Tal Gavriel, 2018). Thereby, FISH is generally not appropriated for studies with a large sample size.

#### Sequencing Techniques

Sequencing techniques, either based on the construction of 16S rRNA gene clone libraries or recent high-throughput methods such as 454-pyrosequencing or Illumina sequencing, have allowed the identification of previously uncharacterized bacterial groups.

Five different bacterial phyla were identified in the stomach and intestines of healthy cats using traditional Sanger sequencing, with sequences predominantly classified in phylum *Firmicutes* (68%), followed by *Proteobacteria* (14%), *Bacteroidetes* (10%), *Fusobacteria* (5%), and *Actinobacteria* (4%) (Ritchie et al., 2008; Minamoto et al., 2012). Most clones belong to order *Clostridiales* (54%), followed by *Lactobacillales* in jejunum and *Bacteroidales* in ileum and colon. However, different percentages of abundance were found in another study that used the 60-kDa chaperonin (cpn60) gene as target. In this study, the most abundant phylum found was *Firmicutes*, followed by *Actinobacteria*, *Bacteroidetes*, and *Proteobacteria* (Desai et al., 2008). Although the Sanger technique was one of the first methods used in human and animal research, this method is laborious and has limited throughput, as amplicons must be cloned into bacteria, with individual bacterial colonies sequenced (Sanger et al., 1977; Tal Gavriel, 2018).

High-throughput sequencing techniques, such as 454-pyrosequencing or Illumina sequencing, are capable of sequencing thousands to millions of base pairs in a short amount of time, allowing for in-depth study of the microbiota and relative quantification of amplicons. *Firmicutes* (92%) and *Actinobacteria* (7.3%) were the most abundant phylum reported in the fecal sample of cats using these techniques (Handl et al., 2011). The phylum *Firmicutes* consisted the predominant class *Clostridia* (65%), *Erysipelotrichi* (13%), and *Bacilli* (9%); the class *Clostridia* was dominated by *Clostridium XIVa* and *XI* and *Ruminococcus*; the class *Bacilli* mostly consisted of the order *Lactobacillales*, which was dominated by genera *Enterococcus* and *Lactobacillus*; the class *Erysipelotrichia* only consisted of the order *Erysipelotrichales*, which mainly comprised genera *Turicibacter*, *Catenibacterium*, and *Coprobacillus* (Handl et al., 2011). Some feline studies reported similar distributions of fecal bacterial groups (Desai et al., 2008; Garcia-Mazcorro et al., 2011). Although high-throughput sequencing techniques have been only used in the last decade, these techniques have their disadvantages as well, since the use of universal bacterial primers may underestimate specific bacteria. Moreover, because of the semi-quantitative abundances, detected dynamics by sequencing methods could not precisely present the actual taxon densities (Props et al., 2017). Additionally, due to its cost, only small numbers of samples were analyzed in most of the studies. Therefore, available information obtained from these studies should be considered with caution.

Next-generation sequencing platforms allow metagenomics approaches (i.e., shotgun genomic sequencing). These approaches allow identifying the genes of host and microbes and thereby are able to assess the functional aspect of the microbiome (Gill et al., 2006). Using 454-pyrosequencing, one study revealed that the predominant phylum of fecal microbiota in cats were *Bacteroidetes/Chlorobi* group (68%), *Firmicutes* (13%), *Proteobacteria* (6%), *Actinobacteria* (1.2%), and *Fusobacteria* (0.7%) (Tun et al., 2012). Another study with the metagenomics analysis platform MG-RAST, reported *Bacteroidetes/Chlorobi* group (36.1%), *Firmicutes* (36.3%), *Proteobacteria* (12.4%), and *Actinobacteria* (7.7%) were predominant phyla (Barry et al., 2010). Afterward, using shotgun 454-pyrosequencing, research showed that the dominant bacterial phyla included *Firmicutes* (36–50%), *Bacteroidetes* (24–36%), and *Proteobacteria* (11–12%) (Barry et al., 2012). Metagenomics approaches can also characterize the expression of microbial genes; the major functional metabolic categories are carbohydrate, protein DNA, and amino acid, respectively accounting for 13, 9, 8, 7, and 6% of the feline metagenome (Tun et al., 2012). Apparently, more research needs to be established to investigate in depth the feline metagenome.

#### Discrepancy During the Characterization

Currently, there are several tools to describe the gastrointestinal microbiota. However, discrepancies of microbial abundance are often observed when using different techniques (Abecia et al., 2010; Minamoto et al., 2012). These differences can be partially attributed to different sensitivities and specificities among the different methods. For instance, different sampling sites and methodologies (DNA extraction protocols or PCR primers) have been used in different sequencing studies (Baker et al., 2003; Zoetendal et al., 2004). The sample size may affect the statistical analyses due to limited power (Tal Gavriel, 2018). Collection and processing methods and storage conditions of samples may also result in alterations in the quality of those samples (Ott et al., 2004; Kieler et al., 2016; Tal et al., 2017). Moreover, the specific characteristics of cat populations need to be considered. Variations in the microbiota composition between conventional and specific-pathogen-free cats have been reported, as well as a variation between indoor and outdoor cats (Desai et al., 2008; Ritchie et al., 2008). Furthermore, the composition of the intestinal microbiota could vary between anatomical sites and between the luminal and mucosa-adherent tissues (Ott et al., 2004). Considering these limitations, researchers should exert caution when interpreting results of different experiments and techniques.

Due to the high diversity of the microbial community, less abundant bacterial groups may escape identification, even when using high-throughput sequencing techniques with broad-range primers (Hoffmann et al., 2016). The additional applications of PCR analysis is needed to detect specific groups with low proportion. Significantly, no optimal DNA extraction protocol or PCR-based identification method exists for accurate characterization of all microorganisms, and therefore, the various methods available should be used complementarily.

### Non-bacterial Composition

Aside from bacteria, the mammalian gut harbors many other microbes including archaea, fungi, viruses, and parasites. The intricate relationships between these organisms, the host, and the bacteria are unclear. Current research using pan fungal primers has revealed several fungal components of the cats’ microbiota. Eukaryote (1%) and fungi (0.02%) have been found in feces of cats (Suchodolski et al., 2008; Handl et al., 2011). A study based on 454-pyrosequencing of 18S rRNA gene identified four fungal phyla in feces of cats; *Ascomycota* (>90%) and *Neocallimastigomycota* (>5%) were the predominant phylum. *Ascomycota* was the only and most abundant fungal phylum, dominated by genera *Saccharomyces* and *Aspergillus* (58.31 and 11%) (Suchodolski, 2011a). Research on viral components in the feline GI tract remains lacking. Shotgun sequencing of viral dsDNA found only one order of *Bacteriophages*, *Caudovirales* (Barry et al., 2010). Di Sabatino (2019) reported that viruses accounted for approximately 0.07% of all sequences, and most of them belonged to the *Caudovirales* order and an additional unclassified order. This accounted for a total of 18 families and 42 genera. In addition, Archaea was reported for the first time in this study. This domain accounted for 0.77% of sequences, consisting of five phyla (*Crenarchaeota*, *Euryarchaeota*, *Korarchaeota*, *Nanoarchaeota*, and *Thaumarchaeota*) and twelve classes.

## Role of GI Microbiota

### Nutrition and Metabolism

In addition to the production of energy by using nutrients, the intestinal microbiota produces many metabolites that may have an effect on the host’s health. For example, carbohydrate fermentation leads to the production of short-chain fatty acids (SCFA) (Ramakrishna and Roediger, 1990), further promoting the process of intestinal gluconeogenesis and supporting the formation of lipids; protein fermentation leads to the production of SCFA, ammonia, and branched chain fatty acids (BCFAs), and some studies have revealed that protein fermentation increases phenolic metabolites in humans, which could affect the host’s health (Windey et al., 2012). However, as strict carnivores, cats have several-fold higher intakes of protein compared to other mammals, which seem to have no issues with the carcinogenic effects of protein fermentation (Rissetto et al., 2011). Additionally, certain bacterial groups in the GI tract also play different specific roles. For instance, *Escherichia coli* (*E. coli*) and *Bacteroides* spp. produce vitamin K_2_ (Ramotar et al., 1984), *Enterococcus* spp. synthesize folate (Camilo et al., 1996), *Lactobacillus* and *Bifidobacterium* spp. contribute to the salvage of bile acids (Ridlon et al., 2006), and *Enterococcus casseliflavus* and *Eubacterium ramulus* promote transformation of polyphenols (Schneider et al., 1999), a group of compounds that may confer a health benefit to the host due to their high antioxidant activities (Lambert et al., 2007).

### Promoting Intestinal Structure and Function

Comparative studies between germ-free and specific-pathogen-free animals indicated the essential role of intestinal microbiota on the development of the GI structure and function (Cario et al., 2007; Lutgendorff et al., 2008; Al-Asmakh and Zadjali, 2015). A damaged intestinal epithelial integrity, a decreased intestinal osmolarity, and fatty acid concentrations were found in germ-free mice in a study by Stappenbeck et al. (2002). This evidence highlighted the importance of the microbiota in the development of the intestinal structure. Despite the lack of studies in cats, it is broadly accepted that the main role of the microbiota might be similar among different mammal species. For instance, an *ex vivo* study in cats demonstrated that SCFAs stimulate contraction of colon longitudinal muscles in cats, implying that bacteria-produced SCFAs could possibly promote colonic motility; this result is similar to the findings in humans (Rondeau et al., 2003).

### Barrier and Protection

Gastrointestinal microbiota promotes colonization resistance, providing a microbial barrier against potential pathogens by competitive exclusion (Brosey et al., 2000; Lawley and Walker, 2013). It also stimulates the host to produce various antimicrobial compounds such as antimicrobial peptides (AMP) (Othman et al., 2008; Hooper, 2009). Microbial metabolites can also induce AMP expression; for example, SCFAs and lithocholic acid were reported to induce expression of LL-37 cathelicidin by different pathways, which serves a critical role in mammalian innate immune defense against invasive bacterial infection (Schauber et al., 2003; Kida et al., 2006; Termen et al., 2008). Research on this field has been traditionally carried out by human medicine groups using rodent models. Whereas the authors are not aware of any publications on this topic regarding cats, similar effects could be assumed.

### Immunomodulation

Gastrointestinal microbiota contributes to gut immunomodulation together with both innate and adaptive immune systems (Suzuki et al., 2010; Geuking et al., 2011; Chung et al., 2012). Studies have shown that probiotic administration can have immunomodulatory effects. For example, the supplementation of *Lactobacillus acidophilus* DSM13241 (2 × 10^8^ CFU/d for 4.5 weeks) in healthy adult cats increased the phagocytic capacity in the peripheral granulocytes and decreased the concentration of endotoxins in plasma (Marshall-Jones et al., 2006).

### Regulation Outside the Gut

A healthy microbial ecosystem is not only important for the GI tract itself. There is growing evidence on the close interaction between the gut microbiota and the body’s major neuroendocrine system, the hypothalamic–pituitary–adrenal (HPA) axis, which controls various body processes in response to stress (Sudo et al., 2004; Sudo, 2006). The role of microbiota on the development of neural processes is being currently studied and termed the “brain-gut microbial axis” (Forsythe et al., 2009; Rhee et al., 2009). This area has not been investigated in cats yet.

### Relationship With Disease

A dysregulated GI mucosal homeostasis has been associated with several diseases (Honda and Takeda, 2009). For instance, IBD often presents itself together with an altered abundance of groups of the feline microbiota, particularly *Enterobacteriaceae* and *Desulfovibrio* (Inness et al., 2007; Janeczko et al., 2008). The GI microbiota plays a vital role on the pathologies that affect the gut, but the relationship remains unclear. For instance, several enteric pathogens, such as *Salmonella*, *Campylobacter jejuni*, *Clostridium perfringens* (*C. perfringens*), and *E. coli*, can also be found in healthy cats (Marks et al., 2011). The microbiota is a contributor to intestinal homeostasis; it is consequently associated with the progress of diseases.

## Factors Influencing Microbiota in Healthy Cats

### Age, Gender, and Neutering

Aging has been associated with a number of changes in the gut of animals. These changes may lead to an increasing incidence of several chronic diseases (Bermingham et al., 2012). A study on the impacts of neutering, neutering age, and gender on the GI microbiota of cats during growth revealed that, when diets and environments are controlled, the only significant association found was age (Deusch et al., 2015). Masuoka et al. (2017) used cultivation as well as real-time PCR to assess feline fecal microbiota in five different age groups (12.6 ± 0.5 days; 7.5 ± 0.5 weeks; 2.5 ± 0.5 years; 11.6 ± 1.6 years; 17.5 ± 1.2 years), finding that the composition of feline fecal microbiota changed with age. The age-related shifts however differed from results from humans and dogs, which involved *Bifidobacteria*, *C. perfringens*, *Lactobacillus*, *Enterobacteriaceae*, and *Enterococcus*. Nevertheless, because this study did not use the sequencing method, the results may not be comparable. In addition, the species composition of *Lactobacillus* was only found in preweaning and young groups but not in aged and senile cats, suggesting that *Lactobacillus* species may be not as important for cat health as in the case of humans and dogs. A similar conclusion was found in another longitudinal study, which did not identify *Bifidobacteria* in young cats but demonstrated similar *Lactobacilli* abundance in cats at different ages (8–260 weeks) using the 16S rRNA gene-based method (Bermingham et al., 2018). In this study, the most dominant group in young cats that were fed kibbled diets was an unclassified *Peptostreptococcaceae* (22.5%), whereas in older cats fed with the same diet, the most dominant group was *Prevotella* (29.7%). The abundance of *Lactobacillus* in older cats was significantly lower compared to that found in young cats.

Although several studies have investigated the impacts of age on the GI microbiota of cats, these studies are often confounded by differences in diet, by individual variation, and by the different methodologies used to analyze the microbiome. Moreover, the results of these studies are often inconsistent, and the impact of gender and neutering remains unclear as well. Nevertheless, these studies have shown that the significance of single component bacteria (e.g., *Lactobacillus* and *Bifidobacteria*) in the GI microbiota in cats may be different from that of dogs or humans. This is an interesting finding that could lead to further research.

### Diet

The nutritional composition of food has been proved to influence the intestinal function, microbial composition, and metabolism (GrzesKowiak et al., 2015). Pet food is highly variable regarding the format (i.e., dry vs. wet, conventional vs. alternative), macronutrient sources and composition, and content of functional ingredients such as prebiotics and probiotics. Several diet-related factors affecting the GI microbiota have been reported by nutritional studies focused on the GI microbiota (summary in Table 2).

**TABLE 2:** Summary of available research on the dietary effects on feline intestinal microbiota.

References	Diet	Method	Alterations of microbiota
Bermingham et al. (2013a)	Preweaning and postweaning diet (*n* = 5 per group)	454-Pyrosequencing	Postweaning diet: *Fusobacteria*↓ *Firmicutes*, *Actinobacteria* ↑
Kerr et al. (2014)	Extruded diets and whole chicks (*n* = 4)	454-Pyrosequencing	Extruded diets: *Faecalibacterium*, *Succinivibrio* ↑
			Chicks: *Lachnospiraceae*, *Peptococcus*, *Pseudobutyrivibrio* ↑
Young et al. (2016)	Kibbled and canned diet (*n* = 5)	Shotgun sequencing	Kibbled diets: *Lactobacillus*, *Bifidobacterium*, *Collinsella* ↑
Lubbs et al. (2009)	Different protein concentration (*n* = 4)	qPCR DGGE	High protein: *Bifidobacterium*↓ *C. perfringens* ↑
Hooda et al. (2013)	Protein: carbohydrate ratio (*n* = 7)	454-Pyrosequencing	Moderate protein and moderate carbohydrate vs. high protein and low carbohydrate: *Actinobacteria* ↑, *Fusobacteria*↓
Barry et al. (2012)	Add 3 prebiotic substances (*n* = 4)	Shotgun sequencing	Fructooligosaccharides: *Actinobacteria* ↑ Pectin: *Firmicutes*, total bacteria ↑
Barry et al. (2014)	Add 2 prebiotic substances (*n* = 6)	qPCR DGGE	Oligofructose + insulin: *Bifidobacteria* spp., *E. coli*↓
Pinna (2014)	Add 6 prebiotic substances and 2 levels protein (*n* = 4)	FISH	Lactitol and pectins: *Enterobacteriaceae*↓
			High protein: *C. perfringens* ↑ *Lactobacillus* spp., *Enterococci*↓
Santos et al. (2018)	Add spray-dried yeast cell wall (*n* = 4)	qPCR	*Bifidobacterium* spp., *Lactobacillus* spp. ↑ *C. perfringens*, *E. coli*↓
Deb-Choudhury et al. (2018)	Add wool hydrolysate, insulin and cellulose (*n* = 8)	qPCR	Wool hydrolysate and cellulose: *Prevotella*, *Bulleidia*, *Faecalibacterium*, *Ruminococcaceae*↓; *Fusobacterium* ↑

#### Type of Diets

A 454-pyrosequencing-based feline study found that cats that were fed a dry diet [32.91% crude protein, 11.05% crude fat, and 1.88% crude fiber; dry matter (DM)] displayed a higher abundance of *Actinobacteria* and lower abundance of *Fusobacteria* and *Proteobacteria* than those fed with wet food (41.87% crude protein, 42.39% crude fat, and 1.62% crude fiber; DM). However, changes on the microbiota composition cannot be attributed to any specific nutrients (Bermingham et al., 2013b). Kerr et al. (2014) compared the fecal bacterial population in cats fed chicken-based extruded diets (38.9% crude protein and 14.4% crude fat, DM) with those fed commercial 1–3-day-old chicks (71.4% crude protein and 20.0% crude fat, DM). Cats fed chicks showed a higher abundance of genera: *Peptococcus*, *Pseudobutyrivibrio*, and unclassified *Lachnospiraceae*, while those fed extruded diets displayed a higher abundance of *Faecalibacterium* and *Succinivibrio*. Furthermore, *Lactobacillus* and *Bifidobacterium* were only detected in cats fed with extruded diets (Kerr et al., 2014). A recent study investigated the impacts of preweaning and postweaning diets (kibbled or canned) in cats using metagenomic sequencing and MG-RAST (Young et al., 2016). In this study, cats fed with kibbled diets (35.3% crude protein, 20.2% crude fat, and 1.8% crude fiber; DM) had higher proportions of *Lactobacillus*, *Bifidobacterium*, and *Collinsella* than those fed with canned diets (45.3% crude protein, 37.6% crude fat, and 1.5% crude fiber; DM). Several metagenomic differences in cats fed with canned diet revealed the related metabolic pathways, associated with vitamin biosynthesis, metabolism, and transport (Young et al., 2016).

Conventional commercial cat foods generally fall into two broad categories: dry (approximately 30–40% crude protein and 30–40% crude fat) or wet (approximately 40–50% crude protein and 40–50% crude fat) (Villaverde and Fascetti, 2014). Furthermore, the consumption of “alternative” pet food, for instance fresh/refrigerated, raw/frozen, and dehydrated varieties of pet food, is becoming a new customer trend (Berschneider, 2002). Studies demonstrated that short-term dietary exposure to diet leads to large shifts in the fecal bacterial population that have the potential to affect the cat’s ability to process macronutrients in the diet, therefore impacting the functional capacities of the microbiota and its interaction with the host. In addition, metagenomic research has suggested that the modulation of the microbiome function through diet may be an important method for improving the nutrition of companion animals. Further research is needed to determine the impact of these shifts on the long-term health of domestic cats and the impact of the newly developed dietary types.

#### Energy Source Composition

Traditional commercial cat food often contains 30–40% of protein (Hooda et al., 2013). However, cats are obligate carnivores, having evolved on diets rich in protein and fat (Plantinga et al., 2011; Depauw et al., 2013); therefore, the content of dietary protein has always been a hot topic in feline research. In a 2009 study, Lubbs et al. (2009) identified several variations in the GI microbiota composition in adult cats that were fed diets with different proportions of proteins. Cats fed moderate protein (MP, approximately 30% crude protein) had a bacterial similarity index (Dice’s similarity coefficient) of 66.7% while those fed high protein (HP, approximately 50% crude protein) had 40.6%. Similarly as in dogs and humans, a higher dietary protein concentration decreased *Bifidobacterium* and increased *C. perfringens* populations (Zentek et al., 2003; Singh et al., 2017). In addition, Bermingham et al. (2013a) showed significant differences in the GI microbiota of pregnant and lactating queens that were fed either a moderate protein:fat:carbohydrate kibbled diet (35:20:28%, DM), a preweaning diet, or a high protein:fat:carbohydrate canned (45:37:2%, DM) postweaning diet. However, the preweaning diet had a low impact on the fecal microbial population of weaned kittens, whereas the postweaning diet changed considerably the microbial community profile. Moreover, kittens fed with the postweaning diet had a lower abundance of *Firmicutes* and *Actinobacteria* and a higher abundance of *Fusobacteria* when compared with those fed the preweaning diet (Bermingham et al., 2013a). Hooda et al. (2013) also showed an increased *Actinobacter* abundance and decreased *Fusobacteria* abundance at the phylum level by moderate protein diet and moderate carbohydrate (34% protein and 19% fat; DM) diet compared with those fed a high-protein, low-carbohydrate diet (53% protein and 24% fat; DM) using 454-pyrosequencing in 8–16-week-old kittens. Using the same samples from Hooda’s study, Deusch et al. (2014) investigated the microbiome function by using shotgun sequencing, revealing a great dietary influence on the pathways associated with amino acid biosynthesis and metabolism.

Many studies have been conducted to investigate the impact of dietary macronutrient concentrations on the GI microbiota of humans and dogs. However, only few studies have studied the effect of dietary carbohydrates/protein/fat concentration in cats. Moreover, it is complicated to reach any accurate conclusions about the types of nutrients that could cause the observed changes in the microbial population, as the macronutrient contents and ingredient composition of diets differed largely in these studies. Furthermore, cats, as obligate carnivores, consume little plant materials under natural conditions; therefore, data collected from adapted carnivores and omnivores, such as dogs and humans, in other nutritional studies cannot be simply extrapolated to cats (Verbrugghe et al., 2012).

#### Supplementation of Fibers

In 2010, Barry et al. (2010) evaluated the effects of dietary supplementation of 4% cellulose, fructooligosaccharides, or pectin on the fecal microbial population in cats. In this study, an increased proportion of fecal *Bifidobacterium* and a decreased proportion of *E. coli* were observed due to the supplementation of fructooligosaccharides. Dietary supplementation of pectin also showed a marked increase in the fecal proportions of *C. perfringens*, *E. coli*, and *Lactobacillus*. The same authors had also performed a similar study using a metagenomic approach and indicated that a 4% fructooligosaccharide supplementation leads to a greater abundance of *Actinobacteria*, while pectin supplementation leads to a greater abundance of *Firmicutes* and total bacteria (Barry et al., 2012). Furthermore, KEGG pathway analysis displayed a strong association between carbohydrates, clustering-based subsystems, protein metabolism, and amino acids and derivatives. In addition, a functional prediction by COG suggested a strong association between amino acid metabolism and transport, and carbohydrate transport and metabolism (Barry et al., 2012). In a different study from the same research group, the effects of fructan supplementation in senior cats were explored. On this occasion, oligofructose or oligofructose + inulin was individually supplemented to the diets at 1% (DM), causing a decrease in *Bifidobacteria* spp. and *E. coli* concentrations (Barry et al., 2014). The results of these studies indicate that, despite the cat being an obligate carnivore, the phylogeny and gene content of its gut microbiome are similar to those of omnivores.

Some studies have attempted to search new functional ingredients for cats, but the alterations of microbiota in current reports are not obvious. An *in vitro* study in 2014 evaluated the influence of different prebiotics and diets with two levels of protein on the fecal microbiota of cats. Supplementation of 2 g/L lactitol and pectin from citrus fruit reduced the count of *Enterobacteriaceae*, and high-protein diets with no supplementation increased the count of *C. perfringens* and decreased the counts of *Lactobacillus* and *Enterococci* (Pinna, 2014). Santos et al. (2018) investigated the impacts of increasing the concentration of the spray-dried yeast’s cell wall (0, 0.2, 0.4, 0.6%) in diets on the fecal bacterial composition of healthy adult cats showing a decrease in *C. perfringens* and *E. coli* and an increase in *Bifidobacterium* and *Lactobacillus* with increasing yeast cell wall. In addition, a dietary supplementation of 2% wool hydrolysate has been recently reported to influence the composition of the fecal microbiota in a similar way as cellulose supplementation (Deb-Choudhury et al., 2018).

The supplementation of fibers has been frequently applied in animal nutrition to improve the quality of the food or the performance and/or health of the animals (Jones and Jew, 2007). Because of the obvious effects modulating the microbial environment, some recent studies have also investigated its potential benefits for cats. Despite the short colon and the lack of a functional cecum as part of evolutionary adaptations to a strict carnivorous diet, considerable microbial fermentation occurs in the hindgut of domestic cats (Verbrugghe et al., 2012). The microbiota of domestic cats has been showed to be capable of fermenting a broad range of dietary fibers, including prebiotics. As a consequence, many attempts have been established to evaluate the effects of fibers in cats. Nevertheless, the shifts of the microbiota in most studies are so small that researchers usually cannot draw accurate conclusions.

#### Future Direction

Numerous studies on GI microbiota have revealed that diets alter the population and functionality of the community of microorganisms in the gut of humans and animals (David et al., 2014). The research on nutritional intervention in the last decade has demonstrated the extent to which the intestinal microbial community could be affected by diet changes (Sonnenburg et al., 2004). This can be summarized as three major aspects: (i) the microbiome rapidly responds to short-term macronutrient changes, (ii) long-term dietary habits are a dominant force in determining the composition of an individual’s gut microbiota, and (iii) a change in the diet has significantly variable effects in different individuals. Regarding the first theme, plenty of attempts to study this response using different designs and multiple techniques have been performed, as listed in the previous sections. However, there are few long-term studies investigating the effect of diet on the feline microbiota; the study on individual variability remains lacking. In addition, different results can be often found within similar designed studies, partially due to the different techniques being employed. These limitations have pointed out a very broad direction for future research. With the current development of metagenomics, it may be necessary to repeat some existing studies that showed marked shifts in the microbial population in order to obtain detailed information about the functional alterations on the microbiota.

### Diseases

Similar to the findings in humans, an unbalanced GI microbiota in cats could lead to gastrointestinal disorders, caused not only by the proliferation of enteropathogens in the GI tract but also by the various metabolic processes in which the GI microbiota participates (Deng and Swanson, 2015). The brief introduction of research progress on the alteration of feline microbiota caused by GI disease, metabolic disease and others is presented in Table 3.

**TABLE 3:** Summary of available research on the intestinal microbiota in cats with diseases.

References	Sample	Disease	Method	Alterations of microbiota
Suchodolski et al. (2015)	Feces	Acute (*n* = 19) or chronic diarrhea (*n* = 29)	Sequencing and qPCR	*Burkholderiales*, *Enterobacteriaceae*, *Streptococcus*, *Collinsella*↑ *Campylobacterales*, *Bacteroidaceae*, *Megamonas*, *Helicobacter*, *Roseburia*↓
Inness et al. (2007)	Feces	IBD (*n* = 11)	FISH	IBD: Total bacteria, *Bifidobacterium spp.*, *Bacteroides*↓ *Desulfovibrio*↑
Janeczko et al. (2008)	Intestine Biopsies	IBD (*n* = 13)	FISH	*Enterobacteriaceae*↑
Fischer et al. (2017)	Feces	Obesity (*n* = 8)	16S rRNA sequencing	*Firmicutes*, *Peptostreptococcaceae*↑ *Bacteroidetes*↓
Pallotto et al. (2018)	Feces	Obesity (*n* = 4)	Illumina sequencing	Restriction of diet: *Actinobacteria*↑; *Bacteroidetes*↓
Ghosh et al. (2013)	Ileum Biopsies	Severe systemic ill (*n* = 50)	FISH	*Enterococcus faecalis*↑
Weese et al. (2015)	Rectum	Immunodeficiency (*n* = 16)	Illumina sequencing	*Bifidobacteriales*, *Lactobacillales*, *Aeromonadales*↑
Rossi et al. (2017)	Feces	Megacolon (*n* = 3) and constipation (*n* = 7)	qPCR	Probiotic (SLAB51^TM^): *Lactobacillus spp.*, *Bacteroidetes*↑
Schmid et al. (2018)	Feces	Severe GI diseases (*n* = 6)	Illumina sequencing	Omeprazole: *Bifidobacterium spp.*↑ *Streptococcus*, *Lactobacillus*, *Clostridium*, *Faecalibacterium spp.*↓
Marsilio et al. (2019)	Feces	IBD (*n* = 13) and SCL (*n* = 14)	Illumina sequencing	*Enterobacteriaceae*, *Streptococcaceae*↑ *Ruminococcaceae*, *Turicibacteraceae*, *Bifidobacterium, Bacteroidetes*↓
Kieler et al. (2019)	Feces	Diabetes mellitus (*n* = 23)	Illumina sequencing	*Anaerotruncus, Dialister, Ruminococcaceae*↓

#### Gastrointestinal Disease

Janeczko et al. (2008) suggested an increase in the number of *Enterobacteriaceae* in the duodenum of cats with IBD, showing a positive correlation between increased microbial counts and the severity of histological inflammation. Cats with IBD also displayed lower counts of total bacteria, *Bacteroides*, and *Bifidobacterium* and a higher count of *Desulfovibrio* compared to healthy cats (Inness et al., 2007). *Desulfovibrio* spp. are sulfate-reducing bacteria that are capable of producing hydrogen sulfides, which might be implicated in the IBD pathogenesis in cats (Janeczko et al., 2008). Nevertheless, in a different study with a similar experimental design, no statistical variation of FISH counts was observed in cats with IBD compared with healthy cats (Abecia et al., 2010). Ramadan et al. (2014) described the alterations of fecal bacterial composition in cats with chronic diarrhea and the effects of diet intervention, finding strong correlations between fecal score and *Coriobacteriaceae*, the *Enterobacteriaceae* family, and an unclassified genus from the order *Clostridiales*. Fecal microbiota in cats with acute and chronic diarrhea showed that, compared to healthy cats, diarrheal cats had an increased fecal microbial abundance of order *Burkholderiales*, family *Enterobacteriaceae*, and genera *Streptococcus* and *Collinsella*. Lower abundance of order *Campylobacterales*, family *Bacteroidaceae*, and genera *Megamonas*, *Helicobacter*, and *Roseburia* was also described (Suchodolski et al., 2015). Moreover, chronic diarrheal cats (>21 days duration) had a higher abundance of class *Erysipelotrichia* and genus *Lactobacillus*, while acute diarrheal cats had a higher abundance of phylum *Bacteroidetes* (Suchodolski et al., 2015). The impact of a multi-strain probiotic (SLAB51TM) was investigated in constipated and megacolon cats. No differences were found in the microbial population of healthy control cats and constipated cats. However, the administration of probiotics markedly increased the presence of *Lactobacillus* and *Bacteroidetes* in constipated cats (Rossi et al., 2017). Using Illumina sequencing, Marsilio et al. (2019) revealed a decreased alpha diversity in cats with IBD and small cell lymphoma (SCL) and a significant difference in bacterial communities with a small effect size. Cats with IBD and SCL both displayed a higher abundance of *Enterobacteriaceae* and *Streptococcaceae* and a lower abundance of *Ruminococcaceae*, *Turicibacteraceae*, *Bifidobacterium*, and *Bacteroidetes* (Marsilio et al., 2019).

Microbiota imbalance, often defined as “dysbiosis,” refers to any disturbance of the normal microbial content that can disrupt the symbiotic relationships between microorganisms and the host, potentially leading to the onset of pathologies (Carding et al., 2015). Recent studies have demonstrated alterations in the GI microbiome due to dietary modifications in cats; only a few studies have evaluated alterations in the intestinal bacterial communities in cats with GI disease. In addition, many of these studies focused exclusively on a particular microbial group using FISH. Furthermore, some of these studies attempted to predict the relationship by correlation analysis, which cannot demonstrate causation.

#### Metabolic Disease

Fecal microbial communities of cats suffering from type 2 diabetes have been indicated to differ from those from healthy cats (Kieler et al., 2019). This new information has raised the interest on the role of environmental factors as the central link between metabolic diseases and the intestinal microbial population (Sonnenburg et al., 2004). Several studies in obese dogs have found a higher abundance of the phyla *Actinobacteria* and *Proteobacteria* and genus *Roseburia*, *Bifidobacteriaceae*, and *Eubacterium*, when compared to fecal microbiota in healthy or lean dogs (Handl et al., 2013; Forster et al., 2018). There are also a few studies that have explored this field in cats.

Bell et al. (2014) attempted to compare the fecal bacterial populations of insulin-treated diabetic and non-diabetic cats, however, no apparent shift was observed in the fecal microbiota composition between diabetic and non-diabetic cats. Using 16S rRNA gene amplicon metabarcoding, Kieler et al. (2019) found that diabetic cats have a decreased gut microbial diversity and a lack of butyrate-producing bacteria genera *Anaerotruncus*, *Dialister*, and unknown *Ruminococcaceae*. Significant differences in fecal bacterial groups were detected between lean cats and overweight or obese cats, but the variations could not be ascribed to the shifts of any particular bacteria (Kieler et al., 2016). Fischer et al. (2017) revealed associations between the shifts in GI microbiota composition and obesity, energy restriction, and neutering. In this study, lean neutered cats displayed a higher abundance of *Firmicutes* and lower abundance of *Bacteroidetes* than the cats in other groups, similarly to previous findings in obese rodents and humans. Additionally, increased *Firmicutes* in lean neutered cats was assigned to the increase in *Peptostreptococcaceae*; decreased fat mass in obese cats by energy restriction was linked to the decreased abundance of genus *Sarcina* and the alterations of a few low-abundance bacterial genera (Fischer et al., 2017). Feeding a moderate-protein and high-fiber diet caused weight loss in overweight cats, resulting in a reduction in *Bacteroidetes* abundance and an increase in *Actinobacteria* abundance (Pallotto et al., 2018). Contrarily, Tal et al. (2020) did not observe significant differences between the fecal microbiome in obese cats during weight loss plan. However, some significantly enriched taxa, mainly belonging to *Firmicutes*, were noted in the linear discriminant analysis effect size test in obese cats before weight loss compared to lean cats (Tal et al., 2020).

#### Others

Ghosh et al. (2013) identified significant differences in the abundance of *Enterococcal* groups in the ileum mucosa between healthy and terminally ill kittens: *E. hirae* was the predominant species of *Enterococci* found in healthy kittens. This species generally lacks virulence traits. Contrarily, *E. faecalis* commonly has several virulence traits and many antimicrobial resistances, while its abundance was found to be higher in the terminally ill kittens. Moreover, a great number of *E. coli* was present in the ileum mucosa of kittens with terminal illness and not detected in all kittens with adherent *E. hirae* (Ghosh et al., 2013). Weese et al. (2015) evaluated the rectal microbial composition of cats infected with feline immunodeficiency virus (FIV); they found that the relative abundance of *Bifidobacteriales*, *Lactobacillales*, and *Aeromonadales* was significantly increased. Schmid et al. (2018) evaluated the impact of prolonged omeprazole administration on the fecal microbiota of healthy cats to identify targets for analysis in a larger subset of cats with GI disease. There was no significant shift of the microbial community and species richness in cats with omeprazole administration. Nevertheless, transient shifts were observed in cats subjected to omeprazole administration, which were associated with a decreased proportion of *Bifidobacterium* and increased proportion of *Streptococcus*, *Lactobacillus*, *Clostridium*, and *Faecalibacterium* (Schmid et al., 2018).

#### Future Direction

A balanced intestinal microbial ecosystem is essential for feline GI health. Profound shifts of GI microbiota have been not only demonstrated in chronic and acute GI diseases but also suggested that those shifts may have a potential role in some extraintestinal diseases (Carding et al., 2015). Nevertheless, most studies have only investigated GI and metabolic diseases and there is little research involving extra-alimentary diseases. Furthermore, the vast majority of recent research on diseases has focused on certain pathogens rather than focusing on the interaction between microbiota and gut. Moreover, many studies have attempted to analyze the relationship between microbiota and disease by using correlations, which cannot demonstrate causation. It would be preferable to design studies aimed at demonstrating a cause–effect relationship or at a minimum acknowledge that in the cases of co-occurring disease and altered microbial status is not possible to identify neither the trigger nor the result. In addition, despite several bacteria being significantly altered, discussion about the size effect is lacking in many papers. The presentation of quantitative effect sizes in addition to *p*-values will improve the ability to perform meta-analysis and to evaluate potentially relevant biological effects. Finally, future research on disease needs to consider how alterations in the microbiota can result in a range of local and systemic diseases. A combination of multiple-omics technologies, such as metagenomics, transcriptomics, and metabolomics, can fill the gap of knowledge in this field. Fortunately, researchers are currently at the initial steps to relate the phylogenetic shift to functional alteration and broaden the knowledge about the biomarker and therapeutic agents of diseases. Future progress on microbiota and diseases is, therefore, to be expected.

## Future Prospect

Research on microbiota in cats benefits of not only the health of the cats themselves but also the health of their owners, since companion animals have the same living environment, similar dietary pattern, and microbial communities as humans (Song et al., 2013). Furthermore, as obligate carnivores, cats have evolved to thrive on a high-protein, high-fat diet; this type of diet is detrimental to humans or other omnivores. Thereby, this unique metabolic pattern associated with microbial activity in cats could serve as a valuable comparative model for the research on the interactions between intestinal microbiota and host metabolism (Eisert, 2011). Moreover, there is some evidence revealed that cats can be a good research model for some human pathologies such as cancer, type 2 diabetes mellitus, and human immunodeficiency virus (Mcniel, 2001; Henson and O’Brien, 2006; Bailey et al., 2010; Bienzle, 2014), all of which have a close connection with the intestinal microbiota. Unfortunately, progress in the study of the feline microbiota has been small compared with the number of studies in humans or other animals, including dogs. This fact could be explained by the lack of funding and the relatively small number of feline research groups. As usual, industry sponsors and specific research foundations will be extremely crucial to the advance and development of this field.

**FIGURE 2:**
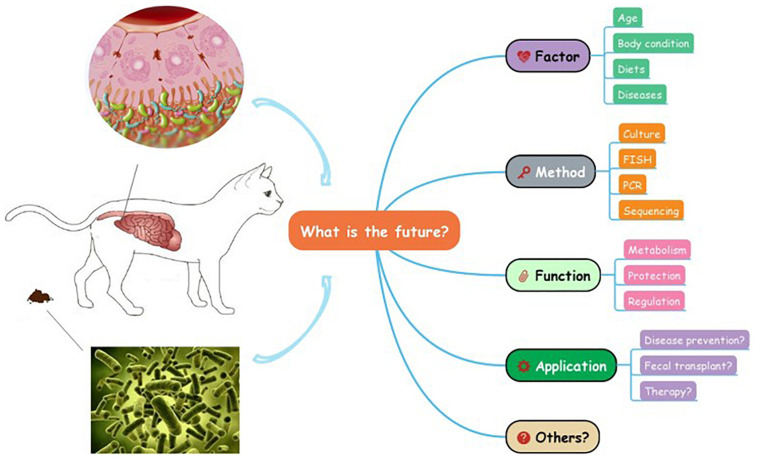
What is the future of the gastrointestinal microbiota in cats?

Great progress has been made in feline research to study the phylogenetic information of GI microbial community, however, the knowledge of microbiota in cats is still at the preliminary stage which can hardly be utilized in practice (Figure 2). In addition, there are some limitations regarding the techniques used in the study of the GI microbiota. For instance, existing sequencing methods often find difficulties identifying the small amounts of microbial DNA present in samples, particularly when the sample sizes are small (Minamoto et al., 2012). Moreover, discrepancies among different analysis tools are warranted to be decreased in the future, and the combined use of multiple techniques and comparisons among different experimental designs can promote progress in this direction. Furthermore, studies in humans and other animals are moving on to combinatorial approaches, such as metagenomics and metatranscriptomics, which could help link microbiome to host health (Quince et al., 2017; Ishii et al., 2018). These new techniques are eagerly anticipated in the future feline studies.

The study of nutrition is one of the most important subjects in life science; however, the vast majority of information on feline nutrition is usually extrapolated from humans. Dietary and metabolic interspecies differences need to be considered; therefore, a database of feline microbiomes, metagenomes, and metabolomes needs to be established. Most of the available research regarding pathologies affecting cats focuses mostly on certain pathogens associated with intestinal conditions rather than the cause/effect relationships between the GI microbiome and the GI health (Marks et al., 2011). To better understand the host–microbe interactions, it is important to study the alterations on the bacterial metabolic function and to investigate the host’s response with the help of metabolomics and transcriptomics. It is also crucial to demonstrate how alterations in the microbiota can result in a range of local and systemic pathologies. The microbiota of cats affected with different diseases, such as diabetes mellitus and feline immunodeficiency virus, should be further researched. Additionally, future applications of microbiota are still expanding in cats. Fecal transplant, for instance, has successfully cured *Clostridioides difficile* infection and IBD in humans and dogs (Gianotti and Moss, 2017; Niina et al., 2019) and ulcerative colitis in cats (Furmanski and Mor, 2017). Finally, all this new information could be applied to design an environmental and dietary strategy and to develop next-generation prebiotics, probiotics, or drugs, which could modulate both the composition and the function of the GI microbial populations and benefit intestinal and host health.

## Author Contributions

YL and CS worked on the original draft preparation. AM worked on the language revision. AV, TV, and MH reviewed the manuscript. All authors contributed to the article and approved the submitted version.

## Conflict of Interest

The authors declare that the research was conducted in the absence of any commercial or financial relationships that could be construed as a potential conflict of interest.

## Footnotes

1 https://scholar.google.com/

2 https://www.ncbi.nlm.nih.gov/

3 https://www.sciencedirect.com/
